# Amalgams

**Published:** 1885-02

**Authors:** W. St. Geo. Elliott

**Affiliations:** London


### AMALGAMS.

BY DR. W. ST. GEO. ELLIOTT, LONDON.

READ BEFORE THE AMERICAN DENTAL SOCIETY OF EUROPE AT ITS TWELFTH ANNUAL MEETING, AT VEVEY, SWITZERLAND, AUG. 26, 27 AND 28, 1884.

Last year I had the pleasure of reading a paper before this society on the subject of Amalgams. I then gave you in tabular form the results of numerous experiments carried on for a period of several months, but I was not satisfied as to their accuracy; my balance turning at a milligramme was not sufficiently delicate, and inadequate time was taken for the experiments. You may remember that I then mentioned that the test I used for shrinkage was that known as the specific gravity test, and that I watched the changes in the specimen under examination for three days.

Thinking to make the experiments more valuable I have gone over the whole field again, and for the specific gravity have used a balance turning at 1-10 of a milligramme. The observations for shrinkage were continued for a week with each kind of amalgam, and the results are given in the table, a part of which was published in the transactions of the Odontological Society of Great Britain.

### SULLIVANS.

| TIME. | DRY. | MEDIUM. | SOFT. |
|---|---|---|---|
| | 1681.5 | 1806.3 | 1810.5 |
| 12 hours,....... | 1703 | 1811.5 | 1817.5 |
| 3 " ...... | 1710 | 1814.3 | 1821.5 |
| 4 " ...... | 1709.2 | 1815 | 1819.4 |
| 4 " ...... | 1710.3 | 1812.2 | 1822.5 |
| 12.45 " ...... | 1713.6 | 1813.5 | 1822.5 |
| 4.15 " ...... | 1715 | 1815.5 | 1822.5 |
| 6.30 " ...... | 1715 | 1812.5 | 1822.5 |
| 13.45 " ...... | 1715.3 | 1811.5 | 1820.2 |
| 4 " ...... | 1710.5 | 1811.5 | 1818 |
| 7.30 " ... .. | 1706.1 | 1808 | 1817.7 |
| 11.30 " ...... | 1706.5 | 1808 | 1817.7 |
| 5 " ...... | 1708.1 | 1808.3 | 1818.2 |
| 7.30 " ...... | 1709.1 | 1808 | 1820 |
| 11.30 " ...... | 1708.9 | 1809 | 1818.6 |
| 6 " ...... | 1709 | 1811 9 | 1827.7 |
| 6 " ...... | 1706.7 | 1809 | 1818.6 |
| 36 " ...... | 1706.7 | 1808.4 | 1820.9 |
| 4.15 " ...... | 1707.5 | 1809.3 | 1820.9 |
| 6.15 " ...... | 1710 | 1807.5 | 1818.5 |
| 7 days, ........ | —29.5 | —1.2 | —8 |

The column on the left is for hours, showing the number of hours since the last observation. The next three columns show the weight in water of the specimen (1 gramme) mixed dry, one medium, and one moist; the dry was mixed with as little mercury as would serve to barely keep it together; the medium, to the consistency ordinarily used in practice, while the moist was very much so. The weights are given in milligrammes, and shrinkage is shown by an increase in weight. The remarkable variations in weight, showing contraction and expansion, have been noticed in all the amalgams experimented on. The last line shows the result in shrinkage at the end of seven days.

| AMALGAMS. | SHRINKAGE. Dry. | SHRINKAGE. Medium. | SHRINKAGE. Soft. | SPECIFIC GRAVITY. Dry. | SPECIFIC GRAVITY. Medium. | SPECIFIC GRAVITY. Soft. | BREAKING STRAIN. Dry. | BREAKING STRAIN. Medium. | BREAKING STRAIN. Soft. |
|---|---|---|---|---|---|---|---|---|---|
| Stewart's Sullivan's,.... | —30.9 | — 5 2 | —13.5 | 9.873 | 12.062 | 10.342 | 2 | 2 9-10 | 2 |
| Sullivan's,.............. | —27.5 | — 1.2 | — 8 | 10.773 | 12.651 | 11.647 | 3 | 3 7-10 | 4 |
| Bonwill's,.............. | —24 | —14.8 | —10.5 | 8.695 | 9.202 | 10.101 | 9 1 5 | 10 2.5 | 6 1-2 |
| Hallam's,.............. | —21.1 | —16.3 | — 7.5 | 8.620 | 9.785 | 9.288 | 17 1-11 | 10 | 3 2-5 |
| Gregory's.............. | — 5 | — 6.3 | —13.9 | 10.127 | 9.478 | 10.476 | 15 7-10 | 10 1-5 | 12 1-10 |
| Lawrence's.............. | —25.5 | — 6.9 | — 5 | 9.083 | 9.174 | 9.523 | 12 1-10 | 9 1-10 | 2 9-10 |
| Flets: Flagg's Stand.,.... | ÷ 2.6 | — 6.6 | — 2 | 9.69 | 9.174 | 9.433 | 15 1-2 | 5 4-5 | 2 9-10 |
| " Extra Plastic,..... | +27 | — 8 7 | — 5.4 | 9.934 | 10.51 | 9.615 | 8 1-10 | 5 3-10 | 5 7-10 |
| " " " 2d qual | —18 | +14 | — 7 | 9.352 | 9.95 | 10.359 | 6 1-5 | 1 3-5 | 1 9-10 |
| Ash's.................. | —10 | — 7 | — 1 | 10.115 | 9.975 | 13.439 | 11 3-5 | 9 1-5 | 10 3-5 |
| Davis' Gold,........ .... | —12 | — 5 | — 8.3 | 9.509 | 9.97 | 10.071 | 9 9-10 | 9 7-10 | 8 1-5 |
| " Crystal Gold, .. | —22.5 | — 8.5 | — 3.5 | 9.345 | 10 | 10.255 | 11 | 10 | 8 4-5 |
| Nickold's,.... .. ....... | —13.4 | — 3 | — 3 | 9.514 | 10.05 | 10.101 | 14 1-11 | 9 4-5 | 8 3-10 |
| Robertson's X. L..,...... | —21 | — 4.4 | — 3 | 9.2165 | 9.756 | 10 | 13 4-5 | 13 3-5 | 11 3-5 |
| Fletcher's Pl. and G.,.... | —23 | —16 | — 5 | 9.0497 | 9.8522 | 10 | 15 7-10 | 14 1-5 | 15 7-10 |
| Robertson's Stand.,...... | — 3 | — 1 6 | +28.4 | 9.2165 | 9.756 | 10.0502 | 15 1-5 | 11 3-5 | 10 |
| Davis' No 2,......... ... | — 7.2 | — 4.5 | +33 | 9.3023 | 9.9502 | 10.1522 | 12 4-5 | 12 1-5 | 11 4-5 |
| Dental M'f'g No. 2,...... | —12.4 | — 3 | — 1.1 | 9.2165 | 9.9502 | 10.2565 | 12 | 11 7-10 | 10 3-10 |
| Fletcher's Flagg's Con.,. | —24 | — 6 | — 3 | 9.4366 | 10.204 | 10.4114 | 9 4-5 | 9 | 12 |
| Standard { Schfeldt and Dubois, | —27 | —11 | — 2 | 9.2639 | 10.2565 | 10.7521 | 10 3-10 | 9 | 12 7-10 |
| Townsend's, ............ | —11 | — 5 | | 9.0497 | 9.756 | 10.1522 | 17 1-5 | 12 | 10 |
| Chicago,................ | | — 2.5 | — 5.5 | 8.5836 | 10 | 10 1011 | 11 7-10 | 9 1-5 | 10 1-5 |
| Arrington's .. .... ..... | +22 | — 3 | + 5 | 8.7719 | 9.9009 | 10 | 11 | 10 | 9 |
| A { Silver, 30 gms...... Tin, 20 " .... } Pure Mercury | —44 | — 4 | — 2 | 8.4786 | 10.2564 | 10.0951 | 9 | 12 | 10 3-5 |
| B { Silver, pure, 15.555 gms.,...... .. Tin, 11.703 gms., .. } Pure Mercury | —14 | — 1 | 0 | 8.4786 | 10.2564 | 10.0951 | 7 | 12 3-5 | 15 1-2 |
| C Coin Silver only,... Pure Mercury | —33 | — 6 | — 7 | 11.7047 | 11.7047 | 11.7047 | 6 1-5 | 8 3-10 | 8 1-5 |
| D { Coin Silver, 15 gms. Tin, 3 gms.,....... } Pure Mercury | —27.6 | —25 | — 5 | | | | 8 | 8 7-10 | 8 1-5 |

The second table gives, first a list of the amalgams experimented upon ; secondly, their aggregate change, shrinkage or expansion in the dry, medium, or wet form ; the next three columns show the specific gravity ; the last three the breaking strain in pounds, as applied to disks, 1 milligramme in thickness and 7.20 milligrammes in diameter ; also in the three forms dry, medium and wet. These disks were made in steel rings of the requisite thickness, and allowed to harden 24 hours ; then the amalgam was extracted from the ring by being placed in a cylinder with an orifice the size of the disk, when a plunger exactly the size of the disk was forced through the cylinder pushing the disk before it; by this means it was possible to remove the disks from the rings without injuring them. After hardening for one month these disks were tested in a micrometer, and were shaved down to 1 mm., tested to the 1-100 of a mm. They were then supported in the breaking apparatus, which consisted of a horizontal cylinder with a blunt conical plunger pressing in the center of the disk, the force being measured by a self-registering balance. I recognize most fully that these experiments cannot be considered conclusive. A much larger number of disks should be made of each kind of amalgam; my opportunities only allowed me to make fifteen to eighteen of each. Perhaps the most prominent benefit the profession can derive from these experiments is the knowledge that there is an individuality about each and every amalgam, which makes it incumbent upon the manufacturers to ascertain positively under what conditions it will give the best results. The majority of practitioners, for some reason, use their amalgam dry; doubtless because in working it gives one the impression that the best results should occur with the least mercury, whereas the experiments go to show that many do better when either mixed medium or moist; some are decidedly better mixed wet. When experiment has proved that a certain proportion of mercury gives the best results, then each package should be so labelled by the maker; we have had enough of the untruthful statements of manufacturers, such as "This Amalgam does not shrink nor discolor in any mouth, etc.," "This Amalgam should be used as dry as possible, etc." The last would be all right if it were followed by some qualifying statement, some facts for us to base our judgment upon. Gentlemen, please to take my experiments *cum grano salis.* Do not accept them as proving positively the property of any amalgam. Indeed, there cannot be that scientific accuracy that we so desire; at least there is no prospect in our time, because it is practically impossible to get perfectly pure metals to experiment with, and as the amount of impurity must vary, so will the results. My investigations, substantiated by clinical observations, fail to show that even the fairly pure materials we may get give any better or more uniform results than the less expensive; nor do the results in these experiments show greater variation than is to be found in the published results of experiments with the alloys for mechanical purposes.

